# Performance Evaluation of Input Power of Diode Laser on Machined Leather Specimen in Laser Beam Cutting Process

**DOI:** 10.3390/ma16062416

**Published:** 2023-03-17

**Authors:** Tamer Khalaf, Muthuramalingam Thangaraj, Khaja Moiduddin, Vasanth Swaminathan, Syed Hammad Mian, Faraz Ahmed, Mohamed Kamaleldin Aboudaif

**Affiliations:** 1 Department of Industrial Engineering, College of Engineering, King Saud University, Riyadh 11421, Saudi Arabia; 2 Department of Mechatronics Engineering, SRM Institute of Science and Technology, SRM Nagar, Chengalpattu District, Kattankulathur 603203, India; 3 Advanced Manufacturing Institute, King Saud University, Riyadh 11421, Saudi Arabia; 4 Department of Mechanical Engineering, College of Engineering, King Saud University, Riyadh 11421, Saudi Arabia

**Keywords:** leather material, laser beam machining, carbonization, material removal rate, kerf width, power diode

## Abstract

Numerous industries, including footwear, handicrafts, and the automobile industry, utilize leather materials. The main goal of this study was to investigate the effect of input power of the diode laser in laser cutting on vegetable chrome tanned buffalo leather to enhance the cutting process. In the present investigation, carbonization, kerf width, and material removal rate (MRR) were taken as performance measures. The diode-based laser beam machining was designed and fabricated with 2.5 W, 5.5 W, and 20 W diode laser to cut vegetable chrome tanned leather. The high-intensity 20 W diode laser produced lower carbonization, lower kerf width, and higher material removal rate compared with the 2.5 W and 5.5 W diodes. This improved performance was due to the adjustable features associated with this diode laser actuation in the form of circular shape with adjustable diameter. A high power with a lower spot size under pulsed mode can produce higher power density. Since a higher power density can establish less interaction time, it produces lower carbonization. Due to the ability of the 20 W diode laser driver to control the beam shape and size, it could produce a lower kerf width and higher MRR. The optimal parameters for cutting chrome vegetable tanned cow leather were a standoff distance of 18 mm, feed rate of 200 mm/min, and duty cycle of 70%.

## 1. Introduction

Leather is a popular choice for clothing, footwear, furniture, and other accessories due to its flexibility and long-lasting nature. Cutting leather the traditional way is a time-consuming operation. Die cutting is a traditional leatherworking technique that uses a press to cut leather into shapes and sizes. It is a versatile and efficient way to produce complex shapes and patterns from leather. Recent years have seen the use of water jets and CNC knife cutting tables when it comes to leather cutting. In industrial operations, it is critical to maintain consistent quality, and laser technology meets this demand. When the laser cutting parameters are properly selected, the cuts are consistent. Moreover, laser technology is a versatile tool for producing a wide range of shapes and designs. It can be used for cutting complex geometries, designing prototypes, and producing small-scale products [1]. Nesting refers to how the parts are arranged on the sheet to ensure the most efficient use of material, while the cutting sequence is the order in which the laser is directed to cut the material. The performance of the laser cutting process is greatly influenced by nesting and cutting sequence. Proper nesting and an ideal cutting sequence are essential for optimizing the laser cutting process and ensuring that it runs as quickly and efficiently as possible. The most difficult two-dimensional cutting stock challenge encountered in the industry is called the leather nesting problem (LNP). This tricky problem involves finding an efficient way to arrange irregular forms into leather hides with varying curves, holes, and quality zones. It requires a combination of skills including geometry and optimization to determine the optimal layout [2]. Leather skins have a variety of textures and patterns on their surfaces. Indeed, a wide variety of quality zones and holes of various sizes and forms may exist. Additionally, their distribution inside the hide varies greatly. The degree of irregularity in the skins’ contours is likewise rather great. As a result, two leather hides are often rather different. The skins of leather are a natural product. Their interior is frequently diverse, and their shape is exceedingly uneven. A leather hide may have holes, flaws, and areas of varying quality [3]. Laser technology remains very costly, despite the fact that its cost is steadily declining; as a result, its usage is only justified if the final product’s quality is significantly improved, and if the process becomes more dependable [4]. Many sectors have seen a significant increase in laser applications in material cutting because it is now feasible to obtain a higher-quality end product while also increasing process reliability [5]. CO_2_ laser cutting and engraving machines are beneficial for leather production, as they produce high-quality products quickly and with improved quality. This makes them ideal for footwear, handbag, and clothing production [6]. Laser cutting technology eliminates the need for design marks and dies, reducing the need for storage space and the disposal of out-of-date materials. This technology is advantageous because it simplifies the production process and reduces costs [7]. Thus, from this perspective, laser cutting systems provide a good contribution to the environment [8]. Diode lasers are utilized by a wide range of experts and in a number of spectroscopic methods in a variety of pure and applied sciences fields [9]. Due to their numerous bene-fits, semiconductor diode lasers are a suitable choice for material processing. They are effective and affordable, in addition to having a variety of uses to meet various demands [10]. Digital microscopy and SEM photography can be used to evaluate the effectiveness of laser finishing as part of the production process. It should be noted that this process is both time-consuming and costly, but necessary for a variety of material components [11]. Diode laser parameters have a significant effect on the surface topography of the leather, and it is possible to achieve different surface textures through laser treatment. Laser technology can be used to modify the surface of processed leather, providing a new way to tailor the material for different applications. [12]. Laser diode arrays (LDAs) increase the power of laser sources to multiple kilowatts while keeping brightness intact, thus broadening the range of applications for this technology [13]. This method makes use of 20 W diode laser radiation [14]. A study examined the effects of using laser power diodes on leather cutting in regard to the environmental implications [15]. Leather material with a thickness of 1.2 mm was used for the cutting trials. The diode laser was more efficient than the CO_2_ laser when cutting leather, as it produced a smaller heat-affected zone [16]. This reduces the risk of damaging the leather and produces a better-quality end product [17]. A study introduced a cutting machine using a newly designed input power of 5.5 W [18]. Laser beam machining (LBM) utilizes heat to melt and vaporize the material, which can be used to cut leather. It is an advanced and unconventional process, which is more efficient than traditional cutting methods [19]. CO_2_ laser cutting machines are a popular choice for cutting leather materials due to their ability to provide a precise cut with high speed and quality. These machines use lasers to heat the material and create a clean, precise cut [20]. Semiconductor diode lasers are important tools in many industries as they are highly efficient and cost-effective, and they can be used for a range of material processing applications [21]. Diode laser is semiconductor device that produce light in a narrow beam. They are becoming increasingly popular in industrial production due to their cost efficiency, low maintenance needs, and improved accuracy compared to traditional tools [22]. Diode laser cutting is considered more efficient than traditional laser cutting, because it requires a lower optical power demand and can cut through materials of varying thicknesses. Additionally, diode laser cutting offers higher precision and faster cutting speeds [23]. The linear polarization of a carbon dioxide laser beam is more effective when cutting thicker materials, while circular polarization is more effective when cutting thinner materials. Furthermore, the angular orientation of the laser beam may also affect the cutting performance, and the optimal angle is dependent on the material being cut [24]. Burning leather biomaterials during machining operations can produce carbon particles, which can negatively affect product quality. This emphasizes the importance of ensuring operator and environmental safety and health, as well as improving product quality, by minimizing the impact of the carbon particles [25].

It was attempted to investigate different input factors on cutting leather specimens using a diode-assisted LBM process. It was inferred that the carbonization has to be further reduced according to an analysis of carbon formation [26]. Within the extensive literature, only a few studies assessed the effects of diode laser technology on machining vegetable chrome tanned leather using an LBM process. It is also essential to analyze the effects of diode laser at different power on quality measures. Thus, different diode laser were used to explore how laser power affects leather cutting quality. In the present investigation, diode-based LBM with 2.5 W, 5.5 W, and 20 W power was fabricated, and its performance was evaluated in terms of quality measures such as carbonization, kerf width, and material removal rate.

## 2. Experimental Methodology

### 2.1. Selection of Leather Specimens

**Figure 1:**
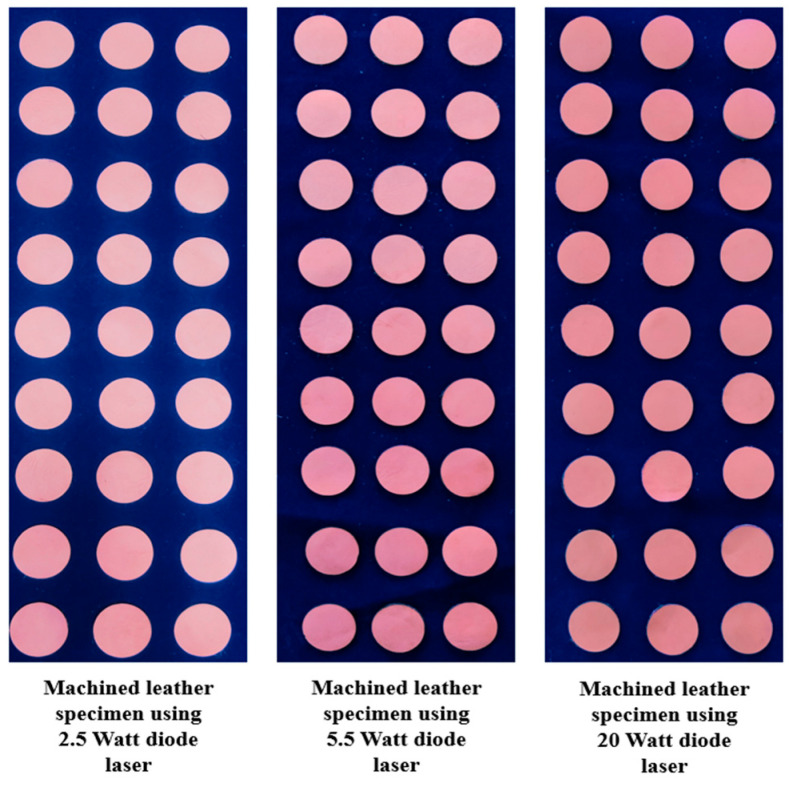
Machined buffalo leather samples.

Figure 1 shows machined buffalo leather samples using 2.5 W, 5.5 W, and 20 W laser power diodes. Vegetable chrome tanned buffalo leather material with a thickness of 1.2 mm was used for the cutting trials. In this investigation, a buffalo leather of 1.2 mm thickness with a shade of light brown was used as a specimen due to its heavy usage. This type of leather is used to manufacture rugged materials such as shoes, wallets, and bags that last a lifetime. Samples were cut to a size of 30 mm × 30 mm.

### 2.2. Design of Laser-Assisted Cutting System

**Figure 2:**
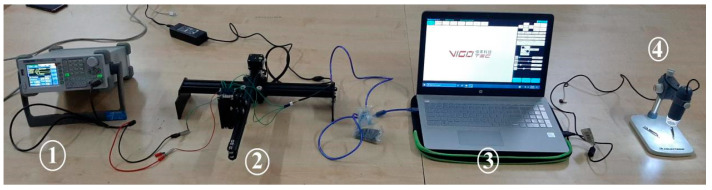
Machined buffalo leather samples under observation using a microscope: (1) waveform generator; (2) LBM setup; (3) software integration; (4) microscope.

The complete laser-assisted cutting system, including all microcontrollers and sensors for measurement, drivers and stepper motors for actuation, and the laser module, is shown in Figure 2, revealing all the systems onboard the machining setup. The block diagram consists of three primary branches that are interdependent of one another for distinct sets of values and readings. The power supply unit (PSU) of 12 V was connected to the laser module, which provided electrical power to the 2.5 W, 5.5 W, and 20 W diodes. A laser driver module was used to control the laser beam. The stepper motors on the *x-* and *y*-axes were driven with the help of the CNC shield through CAM software from a controlling source. A4988 stepper drivers were used to drive the stepper motor. The power supply unit was used to power the A4988 stepper drivers, which operated the NEMA-17 stepper motor used to control the machining system’s *z*-axis, which in turn controlled the standoff distance. Lastly, the power was directed to the Raspberry Pi 4B module, which was responsible for integrating all components and sensors available through an operating system based on the Debian Linux architecture. It was then connected to a 7” touchscreen display, allowing user interaction. This Raspberry PI module also included the open-source computer-aided modeling software “Open Builds CAM”, which was used to control the machining process. The Raspberry Pi module was then linked to an Arduino UNO microcontroller board, which was utilized to drive the *z*-axis stepper motor as a function of the distance from the machining bed, as detected by the time-of-flight VL6180X sensor. Arduino MEGA 2560 is a microcontroller board that runs on the ATmega 2560 CPU. It has 54 digital I/O pins, 16 of which are analog inputs and four of which are UARTs, along with a 16 MHz oscillator, and a DC power supply pin for power supply. The leathers were cut using a 450 nm NEJE 3 Plus N30820 (Manufactured by Zhixinjie Technology Co., Ltd. Shenzhen, China) laser engraver machine. A blue diode laser with input powers of 2.5 W, 5.5 W, and 20 W (Manufactured by Zhixinjie Technology Co., Ltd. Shenzhen, China) was used, as shown in Figure 2. The system on chip (SoC) architecture-based ESP-32 included a Wi-Fi and Bluetooth connection, facilitating wireless integration with a variety of devices.

The Celestron 5 MP CMOS imaging sensor handheld digital microscope (manufactured by Celestron Acquisition, LLC, Torrance, CA, USA) with a 1600 × 1200 pixel array size was used to evaluate the surface quality. MicroCapture Pro software was used to acquire the surface morphology leather cross-section.

### 2.3. Design of Experimental Trials

The standoff distance (SOD), feed rate (FR), and duty cycle (DC) were chosen as the process input parameters to assess the quality of the LBM process. An L_9_ orthogonal array (OA) was chosen for conducting experimental trials as per the Taguchi design, since the present study dealt with three input factors with no interactions among them [10]. The input and output parameters were preferred after considering inputs from various leather suppliers and laser machining specialists. The output variable percentage of carbonization, material removal rate, and kerf width were considered as the output process parameters because of their influence on productivity.

**Figure 3:**
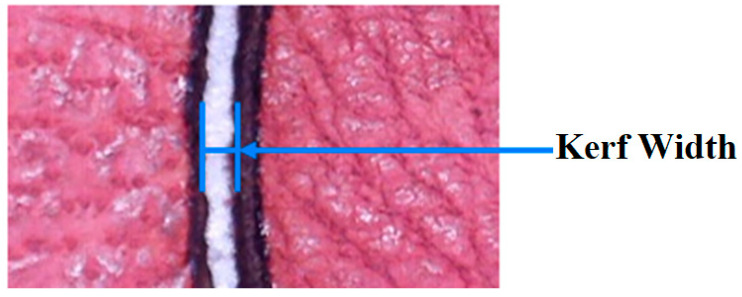
Kerf width analysis in machined leather material.

In leather cutting using the laser ablation technique, the contour edges of the machined leather produce carbon due to the pyrolytic process, since leather is a biomaterial. This process is known as carbonization on leather edges. The percentage carbonization was determined by calculating the number of black and white pixels in the image obtained after the cut, as shown in Figure 3. The image was first converted to grayscale before being converted to black and white using a binary threshold technique [8]. In this experimentation, the carbonization percentage was considered as an output parameter, identified using the Carbonization Analyzer GUI. When pulse width modulation is used, the power intensity of the diode laser can be varied by adjusting the duty cycle. An image processing technique was used to quantify the amount of carbonization in the leather samples. An open-source python library called Open CV-Open computer vision was used to program the algorithm. This library includes all essential codes to achieve the desired outcome. The percentage carbonization was estimated using Equation (1).

(1) $$\mathrm{Carbonization}\;\left(\%\right)=\frac{\mathrm{No}.\;\mathrm{of}\;\mathrm{black}\;\mathrm{pixels}}{\mathrm{No}.\;\mathrm{of}\;\mathrm{black}\;\mathrm{pixels}+\mathrm{No}.\;\mathrm{of}\;\mathrm{white}\;\mathrm{pixels}}\times 100.$$

The material removal rate (MRR) was considered an output parameter to determine the leather material removed per unit time using Equation (2).

(2) $$\mathrm{MRR}=\frac{\mathrm{Weight}\;\mathrm{before}\;\mathrm{cut}-\mathrm{Weight}\;\mathrm{after}\;\mathrm{cut}}{\mathrm{Time}\;\mathrm{taken}}\;$$

Kerf width (KW) indicates the overcut width from laser material removal during the machining process, as indicated in Figure 3.

### 2.4. DEAR Methodology

**Figure 4:**
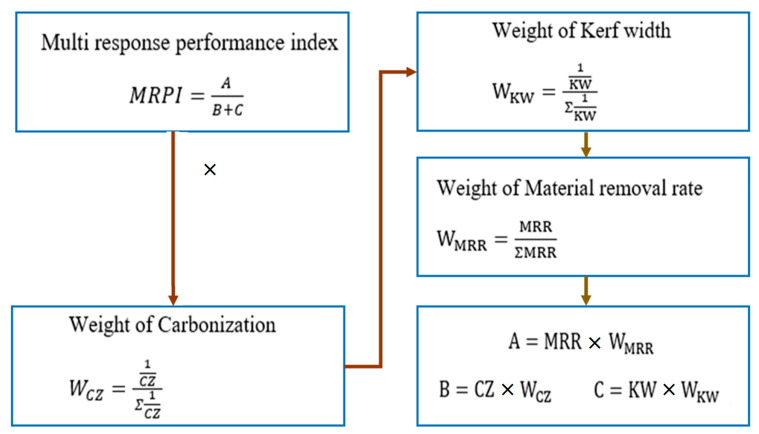
Procedure to calculate MRPI.

Since the conventional Taguchi design can solve only single-response optimization, it is essential to introduce multi-response optimization (MRO). In the present study, Taguchi data envelopment analysis-based ranking (DEAR)-based MRO approach was incorporated owing to its simplicity. Optimization works using a combination of experimental values recorded, which are plotted into a ratio; the following steps are used to optimize the parameters [27]:

- The weights of each response are calculated by determining the fraction between responses over the sum of all experimentation measures.
- These values are further multiplied by their weight to get the weighted data.
- The ratio between “the larger the better” and “the smaller the better”, referred to as the MRPI, was calculated according to the steps shown in Figure 4.
- The average values of carbonization, kerf width, and MRR for the LBM process were taken for the DEAR analysis.

## 3. Results and Discussion

The effects of 2.5 W, 5.5 W, and 20 W diode lasers on performance measures such as carbonization, MRR, and KW are presented in this section. All experimental trials were conducted three times as per the design of experiments, and the average result was taken as the final value to enhance the measurement accuracy.

### 3.1. Effect of Diode Laser on Carbonization under Various Input Powers

Table 1 depicts the carbonization values obtained in the contour edges of the leather material following the LBM process using 2.5 W, 5.5 W, and 20 W diode lasers. It was found that the cutting process using the 2.5 W diode laser had a carbonization range of approximately 72% to 82%, whereas the cutting process using the 5.5 W diode laser had a carbonization range of approximately 58% to 65%. The cutting process using the 20 W diode laser had a carbonization range of approximately 52% to 60%. The duration of laser concentration over the surface was determined by the cutting speed of the laser source movement during the LBM process. Laser power can directly influence the thermal energy produced during the machining process in LBM.

**Figure 5:**
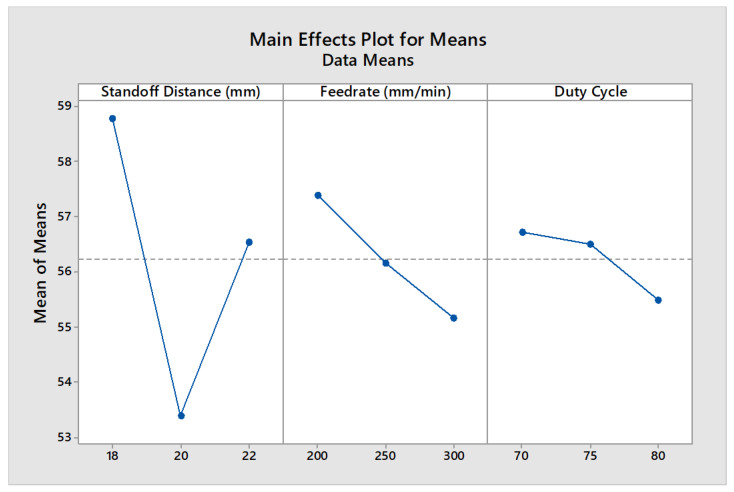
MEP of process factors on carbonization.

A higher thermal energy can quickly vaporize more leather material compared to a lower laser electrical power. However, it was observed that a higher cutting speed with higher laser power could produce a better leather surface quality with a lower carbonization effect. Trial No. 5 produced the optimal level of surface quality with less dross formation. When using the 2.5 W and 5.5 W diode lasers, the laser emits in the form of rectangular shape which cannot be calibrated to a circular shape, whereas, when using the 20 W diode laser, the laser emits in the form of circular shape, and its diameter can be altered using proper signal conditioning of the driver circuit and tuning procedure. A greater cutting surface is required in the case of the 2.5 W and 5.5 W diode lasers due to the presence of rectangular shape, whereas, in the case of the 20 W diode laser, this can be nullified. In the comparison of the 2.5 W, 5.5 W, and 20 W diode lasers while leather machining, it was inferred that the 20 W diode laser produced less carbonization with more adjustable features. The main effects plot (MEP) was used to evaluate the influence of process factors on quality measures, derived using the Minitab 17 software package. Greater aberration from the horizontal mean line indicates a higher influence on the process. It was found that the SOD had a higher influence on carbonization, as inferred from Figure 5.

(3) $$\mathrm{SOD}\;\alpha \;\frac{\mathrm{Peak}\;\mathrm{Pulse}\;\mathrm{Energy}}{\mathrm{Pulse}\;\mathrm{Duration}\times \mathrm{Area}}.$$

(4) $$\mathrm{Power}\;\mathrm{density}\;\alpha \;\frac{\mathrm{Peak}\;\mathrm{Power}}{\mathrm{Area}}.$$

The pulse energy is directly proportional to the SOD, as shown in Equations (3) and (4). A lower SOD can reduce the peak pulse energy, which can reduce the carbonization effect. Dwell time is a crucial factor in diode laser cutting that controls the amount of time the laser beam stays on one spot. It should be adjusted to ensure a quality cut and prevent the material from being exposed to too much heat. Diode laser cutting is an effective way to cut leather without leaving striations, resulting in a smoother finish. The laser beam produced by a diode laser is more precise and controllable than conventional methods, reducing the effects of heat and cutting force on the leather and creating a higher-quality cut. Due to thermal effect, laser cutting causes mild carbonization at the cut edge. Pulse duration is a significant parameter in laser cutting since it regulates the interaction time of the material and energy delivered into the workpiece.

**Figure 6:**
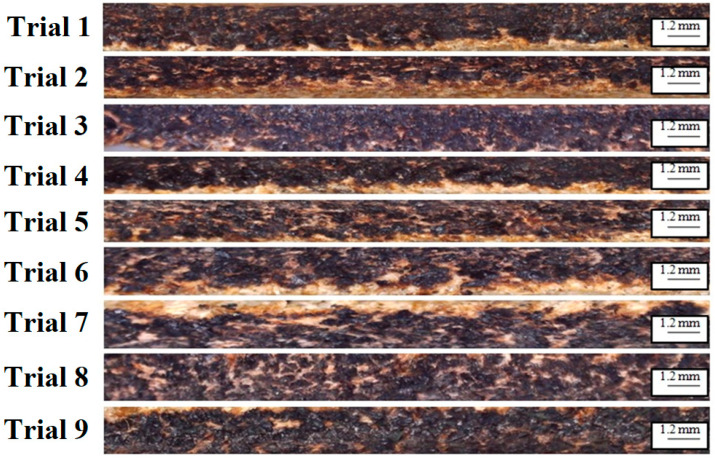
Leather cross-section captured using digital microscope.

The beam and material interaction time is indicated by pulse length, and it is one of the essential factors related to the formation of the heat-affected zone (HAZ). A higher duty cycle can increase the carbonization. The intensity of the laser beam increases with the duty cycle, resulting in enormous heat. The leather is ablated by such heat and fuses again. Figure 6 shows the HAZ region over the machined surface. After machining of the leather specimen using a diode laser, some surplus exertion is induced by the carbonization layer of the machined leather, which can be cleaned with a damp cloth. The machined specimen was inevitably vented after diode laser cutting to eradicate the stench of the flame. The diode-based laser beam machining approach lowered the level of dross in the leather during the cutting operation. The machining method also revealed that no taper creation occurred. Nevertheless, an unwanted carbonization layer was noticed on the outermost surface of the machined surface. The distribution of radiant energy throughout the machining process affected the thickness and form of the carbonization layer.

**Figure 7:**
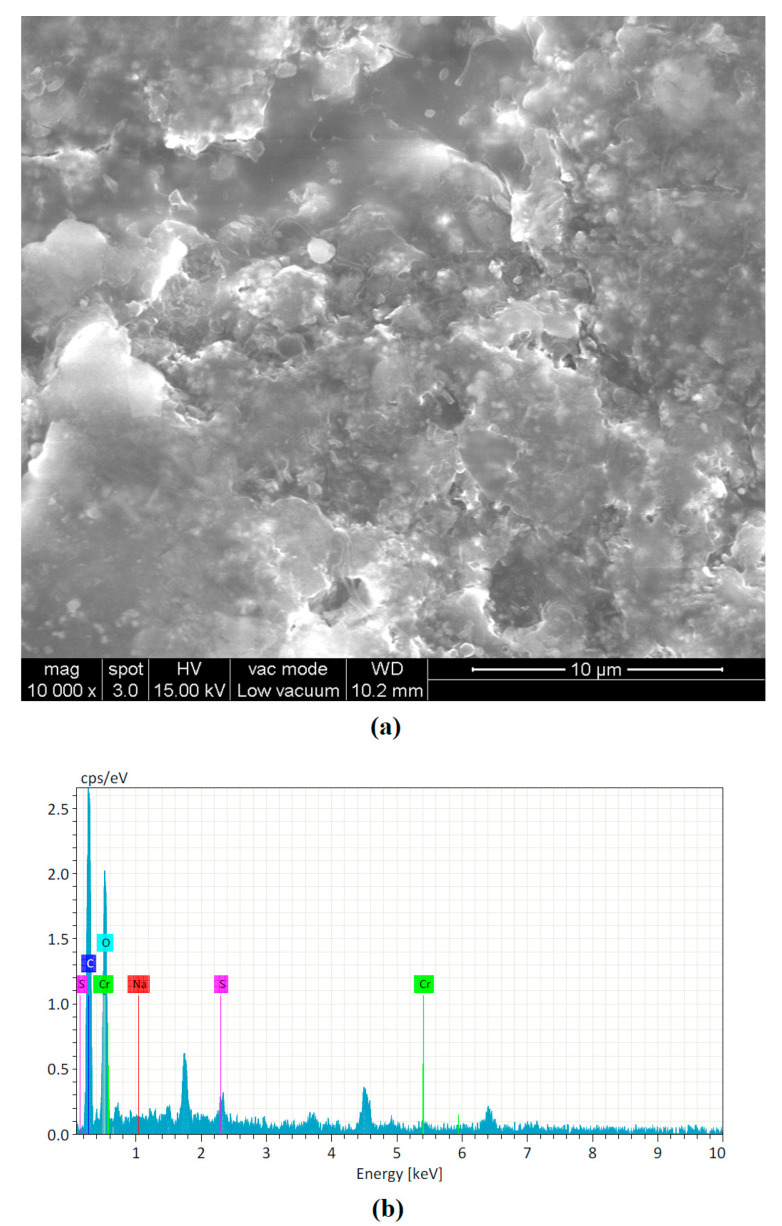
Leather cross-section captured using digital microscope: (**a**) SEM-based surface morphology; (**b**) EDAX analysis.

It was found that Trial No. 5 produced lower carbonization due to a high concentrated energy with moderate standoff distance and feed rate. Trial No. 7 produced a deeper carbonization region owing to a higher standoff distance with moderate duty cycle. A lower feed rate could also produce burnt craters. Figure 7 shows the surface morphology of the cut leather after the LBM process. The HAZ white layer and carbonized black region could be observed after the machining process. Elemental analysis (EDAX) analysis was performed using area mode analysis. It was inferred that the number of carbon particles increased throughout the machining process. 

### 3.2. Effect of Diode Laser on Kerf Width under Various Input Powers

Table 2 depicts the KW values obtained in the contour edges of the leather material following the LBM process using 2.5 W, 5.5 W, and 20 W diode lasers.

**Figure 8:**
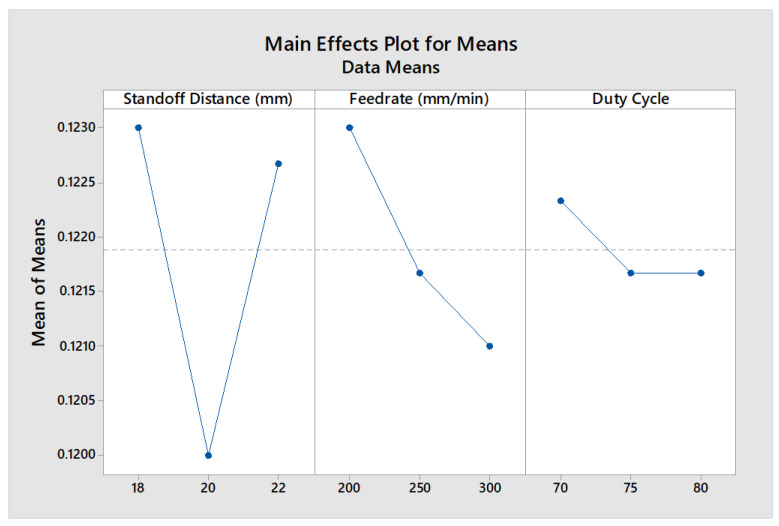
MEP of process factors on KW.

It can be observed that the kerf width ranged from 0.123 mm to 0.128 mm when using the 2.5 W diode laser, whereas it ranged from 0.119 mm to 0.126 mm when using the 2.5 W diode laser. It was observed that the kerf width ranged from 0.119 mm to 0.124 mm when using the 20 W diode laser. As the cutting speed increased, the kerf width decreased, which could occur even at a high laser power. In the comparison of the 2.5 W, 5.5 W, and 20 W diode lasers while leather machining, it was inferred that the 20 W diode laser produced less kerf width since it had an adjustable focal with a circular form. It could be found from the MEP analysis that the SOD had a higher influence on KW, as inferred from Figure 8.

The laser beam generated by the laser is not emitted vertically but has a scattering angle. When using a laser cutting machine to cut material, a certain taper is formed; hence, the quality of the laser beam is also very important in cutting [10]. When the cutting speed increased, the kerf width decreased, and vice versa. With the increase in cutting speed, the dross formation of the leather surface decreased, whereas, with the increase in cutting speed, the machined leather surface improved. However, too great a cutting speed may not achieve penetration; thus, it is very important to control the speed. Among the factors influencing the processing quality and capacity of a laser cutting machine, the most influential is the focus position. The focus position affects processing parameters such as width and cutting speed. This is because a change in the focus position causes a change in the beam diameter on the surface of and incident angle into the processing material. As a result, it influences the formation state of the kerf, as well as the multiple reflections of the beam in the kerf.

### 3.3. Effect of Diode Laser on Material Removal Rate under Various Input Powers

Table 3 depicts the MRR values obtained in the contour edges of the leather material following the LBM process using 2.5 W, 5.5 W, and 20 W diode lasers.

**Figure 9:**
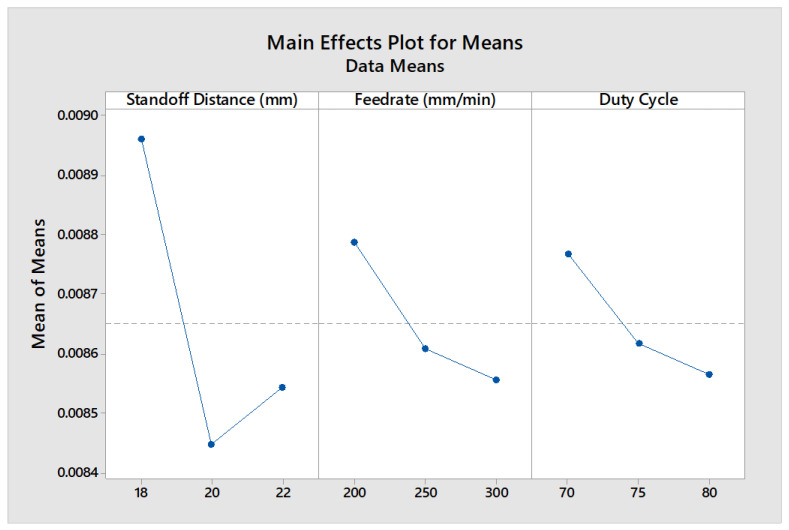
MEP of process factors on MRR.

It can be observed that the 2.5 W diode laser took an average of 62–68 s in cutting a circular shape of leather with a diameter of 30 mm. Thus, the MRR values ranged from approximately 0.006 to 0.007 mg/s, whereas the 5.5 W and 20 W laser diodes could yield MRR values ranging from 0.007 to 0.008 mg/s and from 0.008 to 0.009 mg/s, respectively. In the comparison of the 2.5 W, 5.5 W, and 20 W diode lasers while leather machining, it was inferred that the 20 W diode laser had the maximum material removal rate since it had a higher power intensity. It was found from the MEP analysis that the SOD had a higher influence on MRR, as inferred from Figure 9.

High-quality laser cutting depends on the minimization of unwanted side-effects, such as the heat-affected zone (HAZ) and dross formation. It is well known that these side-effects increase with pulse duration. The material removal rate is defined as the amount of material removed from the workpiece per unit time, which can be calculated from the volume of material removal or the weight difference before and after machining. The MRR is an indication of the machining rate, depending greatly on the process parameters. A higher duty cycle and lower values of pulse interval and pulse duration can result in a higher MRR. The MRR is directly proportional to the feed rate and pulsed frequency of the laser.

### 3.4. Multi-Response Optimization (MRO) of Diode Laser -Assisted Cutting Process Parameters

Although combined control can provide better quality measures than existing approaches, Taguchi DEAR-based MRO was introduced to compute the optimal process parameters to further improve the cutting measures. The weight and MRPI values for each experimental result were computed using the proposed approach, as presented in Table 4.

**Figure 10:**
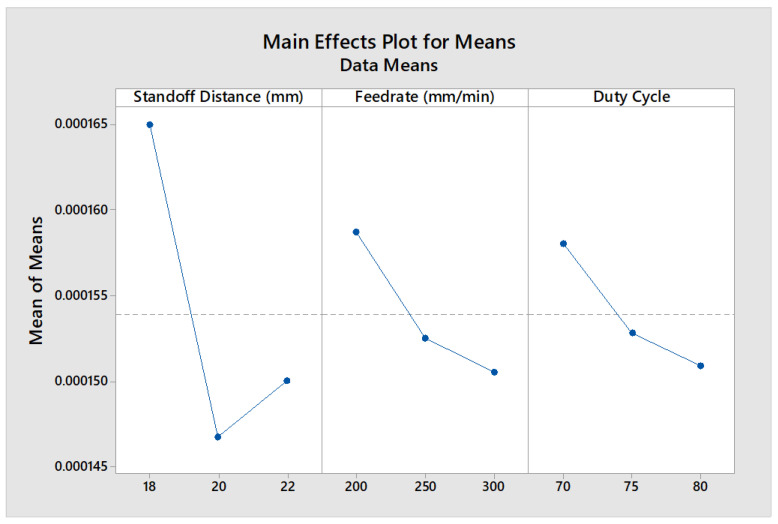
MEP of process factors on MRPI.

The values were computed by including values of MRPI for the corresponding level of each technological parameters. A larger value of MRPI indicates an optimal technology parameter; accordingly, SOD (Level 1), FR (Level 1), and DC (Level 1) resulted in better quality measures, as shown in Table 5, representing the optimal combination of process parameters. According to the DEAR method, it was concluded that the optimal parameters for cutting chrome vegetable tanned cow leather were a standoff distance of 18 mm, feed rate of 200 mm/min, and duty cycle of 70%, as shown in Table 6. To confirm the optimization approach, a confirmation experiment was performed. The experimental results in the optimal conditions showed a 2.3% deviation of the MRPI of the quality indicators compared with the maximum mean value, which is lower than the acceptable tolerance of 5%. Hence, the prediction accuracy of the present approach fell within the acceptable limit. A higher max–min value indicates a more significant influence on the determination of quality parameters in the machining processes. Thus, the standoff distance was identified as the most influential factor in the process due to its importance in determining the power density and peak pulse energy, as per Equations (3) and (4). This was confirmed by the MEP analysis, as shown in Figure 10.

## 4. Conclusions

In the present investigation, performance measures such as carbonization, kerf width, and material removal rate were taken into consideration. An LBM process was designed and fabricated using 2.5 W, 5.5 W and 20 W diode laser to cut vegetable chrome tanned leather. The following conclusions could be drawn from the experimental analysis:

➢ A high power with a lower spot size under pulsed mode can produce a higher power density. Since a higher power density can establish less interaction time, it produces lower carbonization.
➢ Due to the ability of the 20 W diode laser driver to control the beam shape and size, it could produce a lower kerf width and higher MRR.
➢ The high-intensity 20 W diode laser produced a 2.8% lower carbonization, 1.6% lower kerf width, and 2.4% higher MRR than the other diode laser; this was due to its more adjustable features, whereby the laser emitted in the form of a circular shape and its diameter could be altered.
➢ Carbonization can be reduced using a diode laser due to its effect on power density and peak pulse energy.
➢ The optimal parameters for cutting chrome vegetable tanned cow leather were a standoff distance of 18 mm, feed rate of 200 mm/min, and duty cycle of 70%.
➢ The monitoring and control of the diode-assisted LBM process can be performed in a future study.

## Figures and Tables

**Table 1:** Effect of diode laser on carbonization.

Trial No.	Standoff Distance (mm)	Feedrate (mm/min)	Duty Cycle (%)	Carbonization (%)		
				2.5 W	5.5 W	20 W
1	18	200	70	81.917	64.253	59.980
2	18	250	75	80.697	61.840	59.563
3	18	300	80	79.270	62.333	56.810
4	20	200	75	74.560	60.793	54.673
5	20	250	80	72.630	58.190	52.117
6	20	300	70	73.713	59.623	53.387
7	22	200	80	76.383	63.537	57.527
8	22	250	70	75.963	62.167	56.803
9	22	300	75	74.580	60.780	55.283

**Table 2:** Effect of diode laser on kerf width.

Trial No.	Standoff Distance (mm)	Feedrate (mm/min)	Duty Cycle (%)	Kerf Width (mm)		
				2.5 W	5.5 W	20 W
1	18	200	70	0.128	0.126	0.124
2	18	250	75	0.126	0.125	0.123
3	18	300	80	0.127	0.125	0.122
4	20	200	75	0.125	0.123	0.121
5	20	250	80	0.124	0.122	0.119
6	20	300	70	0.124	0.120	0.120
7	22	200	80	0.128	0.125	0.124
8	22	250	70	0.126	0.124	0.123
9	22	300	75	0.125	0.124	0.121

**Table 3:** Effect of diode laser on MRR.

Trial No.	Standoff Distance (mm)	Feedrate (mm/min)	Duty Cycle (%)	Material Removal Rate (mg/s)		
				2.5 W	5.5 W	20 W
1	18	200	70	0.007012	0.008223	0.009015
2	18	250	75	0.006989	0.008094	0.009007
3	18	300	80	0.006937	0.007899	0.008859
4	20	200	75	0.006898	0.007557	0.008631
5	20	250	80	0.006521	0.007218	0.008120
6	20	300	70	0.006733	0.007359	0.008592
7	22	200	80	0.006849	0.007681	0.008716
8	22	250	70	0.006699	0.007608	0.008697
9	22	300	75	0.006601	0.007507	0.008216

**Table 4:** Calculated values for weight and MRPI.

Trial No.	Weights			MRPI
	Carbonization (%)	KW (mm)	MRR (mm/min)	
1	0.10401	0.10920	0.11580	0.00016697
2	0.10473	0.11009	0.11569	0.00016668
3	0.10981	0.11099	0.11379	0.00016124
4	0.11410	0.11191	0.11086	0.00015305
5	0.11970	0.11379	0.10430	0.00013547
6	0.11685	0.11284	0.11036	0.00015167
7	0.10844	0.10920	0.11195	0.00015608
8	0.10982	0.11009	0.11171	0.00015540
9	0.11284	0.11191	0.10553	0.00013869

**Table 5:** Computation of optimal parameter combination.

Factors	Levels			Max–Min
	1	2	3	
SOD	0.0001647	0.0001467	0.0001501	0.00001801
FR	0.0001587	0.0001523	0.0001505	0.00000817
DC	0.0001580	0.0001526	0.0001509	0.0000071

**Table 6:** Optimized process parameters.

Factors	Level	Parameters
SOD	1	18 mm
FR	1	200 mm/min
DC	1	70%

## Data Availability

Not applicable.
